# Transcriptome Analysis of Linalool-Induced Hypopigmentation in B16 Melanoma Cells

**DOI:** 10.3390/ijms27208956

**Published:** 2026-10-09

**Authors:** Myra O. Villareal, Hiroko Isoda

**Affiliations:** 1 Faculty of Science and Technology, Sophia University, Chiyoda City 102-8554, Tokyo, Japan; mvillareal@sophia.ac.jp; 2 Institute of Life and Environmental Science, University of Tsukuba, Tsukuba City 305-8572, Ibaraki, Japan; 3 Alliance for Research on the Mediterranean and North Africa (ARENA), University of Tsukuba, Tsukuba City 305-8572, Ibaraki, Japan

**Keywords:** linalool, antioxidant, B16 melanoma cells, melanogenesis, bioactives, nutraceuticals, natural product

## Abstract

Plant-derived phytochemicals exhibit significant pharmacological potential in chronic diseases, including melanoma. Since hyperactive melanogenesis is closely linked to melanoma progression, targeting this pathway with natural bioactive agents represents a major therapeutic strategy. The plant-derived terpene linalool induces cancer-specific oxidative stress in colon cancer cells, as we have previously reported. Here, we characterized the effects of linalool on B16-F10 melanoma cells to establish a transcriptomic baseline of hypopigmentation networks using phenotypic assays and high-throughput RNA-seq profiling. Non-toxic doses of linalool significantly inhibited melanin synthesis, accompanied by a marked reduction in tyrosinase protein abundance. RNA-seq identified 31 significantly upregulated and 48 significantly downregulated genes (*p* < 0.05), with PCA and cluster analysis demonstrating a distinct transcriptomic profile. Functional GO enrichment revealed candidate regulatory networks involving lysosomal components, the neurofilament cytoskeleton, and cell-cycle checkpoints. While core melanogenesis transcripts (*Mitf*, *Tyr*, *Dct*) showed only minor, non-significant variations, key stress-adaptation and cell-plasticity regulators (*Mir210*, *Dll1*) were significantly suppressed. This profile indicates a state of latent molecular vulnerability, mirroring the oxidative responses in colon cancer cells. Taken together, these findings provide a foundational transcriptomic repository of linalool-responsive networks, uncovering critical candidate pathways that modulate melanin production and antioxidant defenses to guide future functional validation and therapeutic exploration of botanical bioactives in melanoma research.

## 1. Introduction

Natural products are a rich source of bioactive compounds with diverse applications in pharmacological therapeutics, as well as functional foods and cosmeceutical formulations [1,2]. Among these, linalool (2,6-dimethyl-2,7-octadien-6-ol) is a major constituent of essential oils from the Lamiaceae, Lauracea, and Rutaceae families [3]. Found abundantly in plants such as *Lavandula angustifolia* and *Ocimum basilicum* [4,5,6], linalool is prized for its rapid absorption, efficient metabolism, and favorable safety profile [3,7,8]. It is rapidly absorbed via the intestinal tract and efficiently metabolized by the liver into polar compounds for excretion [8]. Linalool exhibits a wide array of pharmacological properties, including sedative, anxiolytic, analgesic, anticonvulsant, and anti-inflammatory activities [9,10,11,12]. Critically, it also possesses significant antioxidant activity, and studies have shown that linalool can scavenge free radicals, reduce hydrogen peroxide-induced oxidative damage, and protect against ultraviolet-light B (UVB) exposure [13,14]. 

We have previously reported that linalool exerts a potent anticancer effect against human colon cancer cells by inducing cancer-specific oxidative stress through the generation of hydroxyl radicals [15]. However, in different cellular environments, linalool functions as an anti-oxidant and modulator [16]. The regulation of this environment is particularly critical in the context of melanogenesis. While melanin production is an essential adaptation for photoprotection, its synthesis is a double-edged sword. The biochemical pathway, governed by the rate-limiting enzyme tyrosinase, involves the oxidation of L-tyrosine and the subsequent formation of reactive quinone intermediates [17]. Paradoxically, while melanin protects against UV radiation, intermediates or variations in cellular homeostasis can generate reactive oxygen species (ROS), where excessive ROS can further accelerate melanogenesis. This creates a vicious cycle of oxidative stress linked to the genomic instability that drives melanoma progression and malignancy [18]. Consequently, the use of potent antioxidants to suppress hyper-melanogenesis has become a cornerstone of anti-melanoma research and pigmentary disorder treatment [19]. 

Although recent studies have documented the inhibitory effects of linalool-rich essential oils on pigmentation, these effects are often confounded by the synergistic actions of multiple chemical constituents [20]. The specific molecular mechanisms by which isolated linalool, a prominent natural bioactive compound, influences the melanogenic pathway, particularly through its transcriptomic regulation of the redox state, remain poorly defined. Given that oxidative stress is a primary catalyst for tyrosinase activity [21], we hypothesized that the antioxidant capacity of linalool is associated with the downregulation of pathways that promote melanogenesis. 

This study was conducted to establish a comprehensive transcriptomic baseline of the hypopigmentation networks induced by linalool in B16 melanoma cells using high-throughput RNA-sequencing (RNA-seq). Through integrated bioinformatic profiling, the global transcriptomic shifts and target gene networks altered by linalool were mapped, revealing candidate regulatory tracks within redox-associated pathways and lysosomal components. Ultimately, this work provides the first global transcriptomic characterization of linalool-responsive networks in melanoma cells, establishing a valuable baseline repository that uncovers novel molecular targets to guide future functional validation and therapeutic exploration of botanical nutraceuticals in melanoma research.

## 2. Results

### 2.1. Linalool Inhibits Melanogenesis

**Figure 1:**
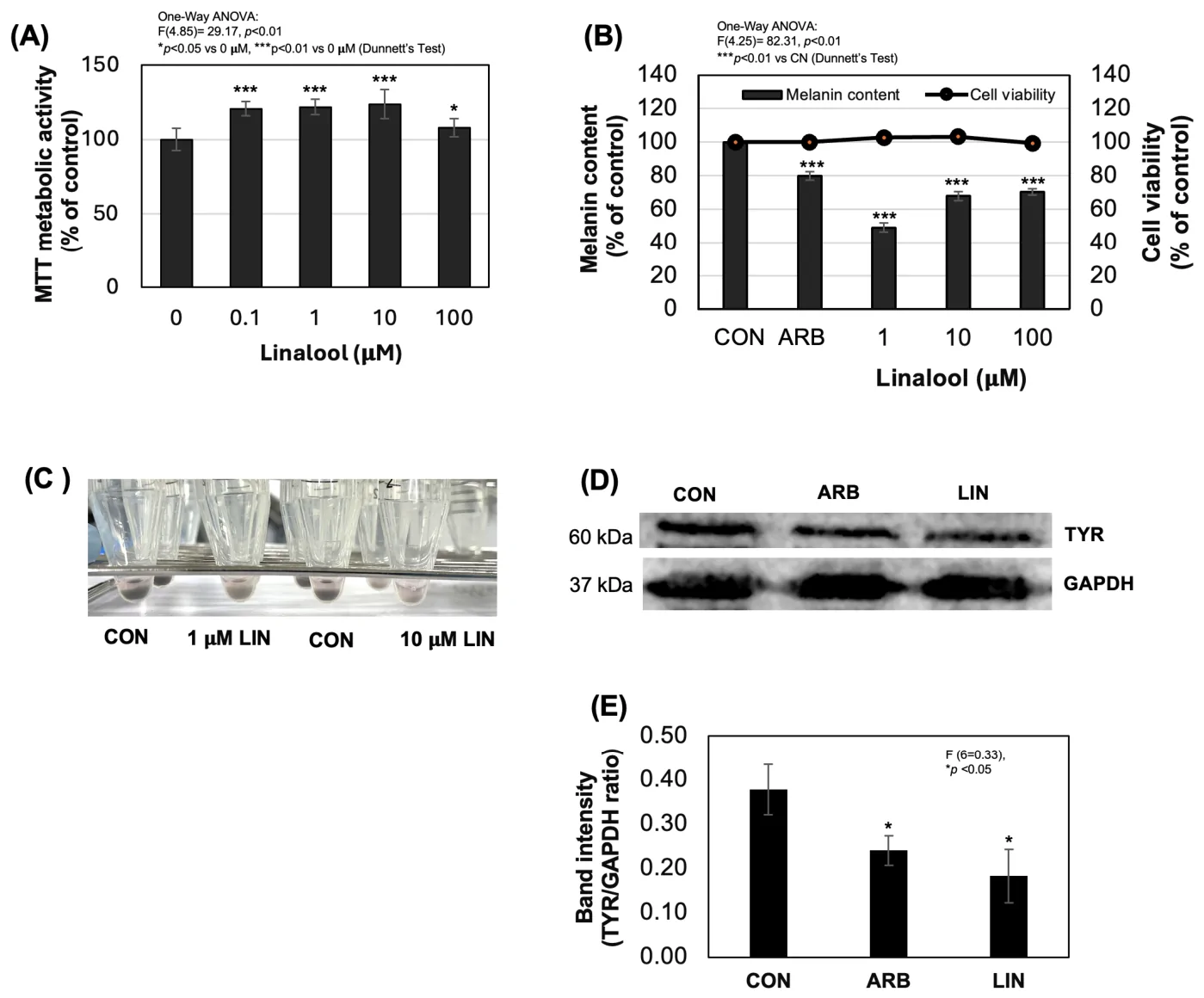
Phenotypic effects of linalool on B16 melanoma cells. (**A**) MTT assay results following 24 h of linalool (LIN) treatment (0.1 to 100 μM) compared to the untreated control. (**B**) Relative melanin content (% of control) alongside the positive control arbutin (ARB). (**C**) B16 melanoma cell lysates showing decreased melanin content in treated cells. (**D**) Representative Western blot analysis showing tyrosinase (TYR) protein levels. (**E**) Densitometric intensity ratio of TYR to GAPDH determined via ImageJ analysis, in ARB and LIN treatment groups relative to the control (CON). Data are expressed as mean ± standard deviation (SD) from three independent experiments. Following verification of normal data distribution, statistical analyses for MTT assay (**A**), relative melanin content (**B**), and the Western blot band intensity ratio (TYR/GAPDH) were performed using a one-way ANOVA to determine overall statistical significance (*p* < 0.05), followed by Dunnett’s post hoc test to compare each linalool treatment group against the untreated control, with independently harvested wells serving as the experimental unit across independent biological replicates (*n* = 3).

To evaluate the functional effects of linalool on B16 melanoma cells prior to transcriptomic profiling, we analyzed cell proliferation, melanin synthesis, and tyrosinase protein level (Figure 1). The MTT assay demonstrated that 24 h of linalool treatment across a concentration range of 0.1 to 100 μM did not induce cytotoxicity. Instead, a global one-way ANOVA revealed a highly significant effect of treatment on MTT metabolic activity [*p* < 0.0001]. Subsequent Dunnett’s post hoc analysis confirmed a statistically significant increase in mitochondrial metabolic activity compared to the untreated control group across all tested concentrations, 0.1 μM (*p* < 0.001), 1 μM (*p* < 0.001), 10 μM (*p* < 0.001), and 100 μM (*p* < 0.05), indicating a modest induction of MTT metabolic activity (Figure 1A). This lack of toxicity was further confirmed by a stable cell viability profile that remained near 100% across all treated groups.

For the melanogenesis assay, a global one-way ANOVA similarly showed a highly significant treatment effect on melanin accumulation [SF(4,25) = 82.31, *p* < 0.0001]. Dunnett’s post hoc comparison against the untreated control (CON) group demonstrated that 1 μM of linalool reduced melanin content of approximately 50% of control levels (*p* < 0.0001), demonstrating a stronger inhibitory effect than the standard positive control arbutin (~80%, *p* < 0.001). Meanwhile, the 10 μM and 100 μM doses maintained a significant suppression at about 70% of control levels (*p* < 0.001; Figure 1B). This phenotypic response showing optimal non-linear efficacy directly guided our selection criteria for subsequent global analysis. This decrease in melanin was visually confirmed by the notable clearing of the corresponding cell lysates (Figure 1C). To investigate the molecular network associated with this depigmenting effect, we performed Western blot analysis on the tyrosinase (TYR), the rate-limiting enzyme of melanogenesis. Both arbutin and linalool treatments markedly decreased tyrosinase protein level relative to the untreated control (Figure 1D,E). 

### 2.2. Overview of the Transcriptome Changes in B16 Cells Induced by Treatment with Linalool 

**Figure 2:**
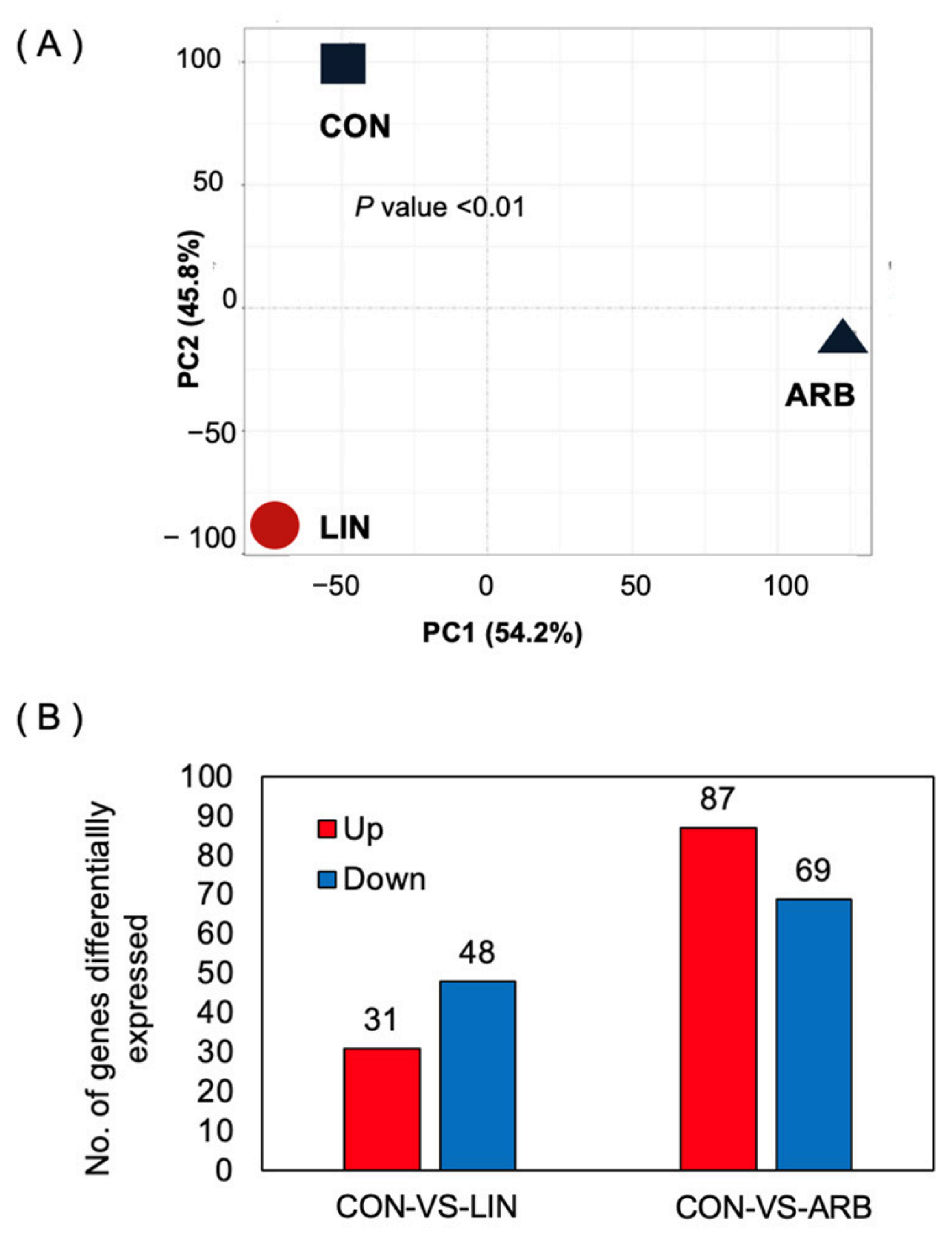
Global transcriptomic profiling and differential gene expression analysis. (**A**) Principal Component Analysis (PCA) score plot demonstrating clear, distinct spatial clustering and variance separation among the control (CON), linalool-treated (LIN), and positive control arbutin (ARB)-treated experimental groups. PC1 accounts for 54.2% of the total variance, and PC2 explains 45.8% of the variance. (**B**) Total number of differentially expressed genes in the linalool-treated group compared to the control group, identifying 31 significantly upregulated and 48 significantly downregulated candidate transcripts. Statistical significance and false discovery rates for differential expression were determined using the Benjamini–Hochberg adjusted Wald test within the DESeq2 pipeline.

To establish a comprehensive transcriptomic baseline of the candidate networks altered by linalool treatment in B16 melanoma cells, we performed a global RNA-seq analysis. The initial spectrophotometric measurement of the isolated RNA yielded A_260_/A_230:_ 0.69–0.89. Based on these initial data indicating potential salt or solvent carryover, the isolated RNA was subjected to an additional cleanup step to remove residual contaminants. The final quality of the purified RNA was subsequently validated, showing an optimal A_260_/A_280_ ratio: 1.98–2.01 ratio range of 1.98–2.01. This successful purification protocol was further corroborated via agarose gel electrophoresis (Supplementary Figure S1), which demonstrated high-purity, intact ribosomal bands with no evidence of degradation. Principal Component Analysis (PCA) demonstrated clear, distinct clustering among the control, linalool-treated, and arbutin-treated groups (*p* < 0.01), Figure 2A). The first principal component (PC1) accounted for 54.2% of the total variance, separating the arbutin group from the control and linalool groups. The second principal component (PC2) explained 45.8% of the variance, sharply distinguishing the linalool-treated cells from both the control and arbutin conditions. In total, the differential expression analysis identified 31 significantly upregulated genes and 48 significantly downregulated candidate transcripts in the linalool-treated group compared to the control (Figure 2B, CON-VS-LIN). For comparison, the positive control arbutin-treated group relative to the control (CON-VS-ARB) yielded 87 upregulated and 69 downregulated transcripts. 

### 2.3. Gene Ontology (GO) Enrichment Analysis 

**Figure 3:**
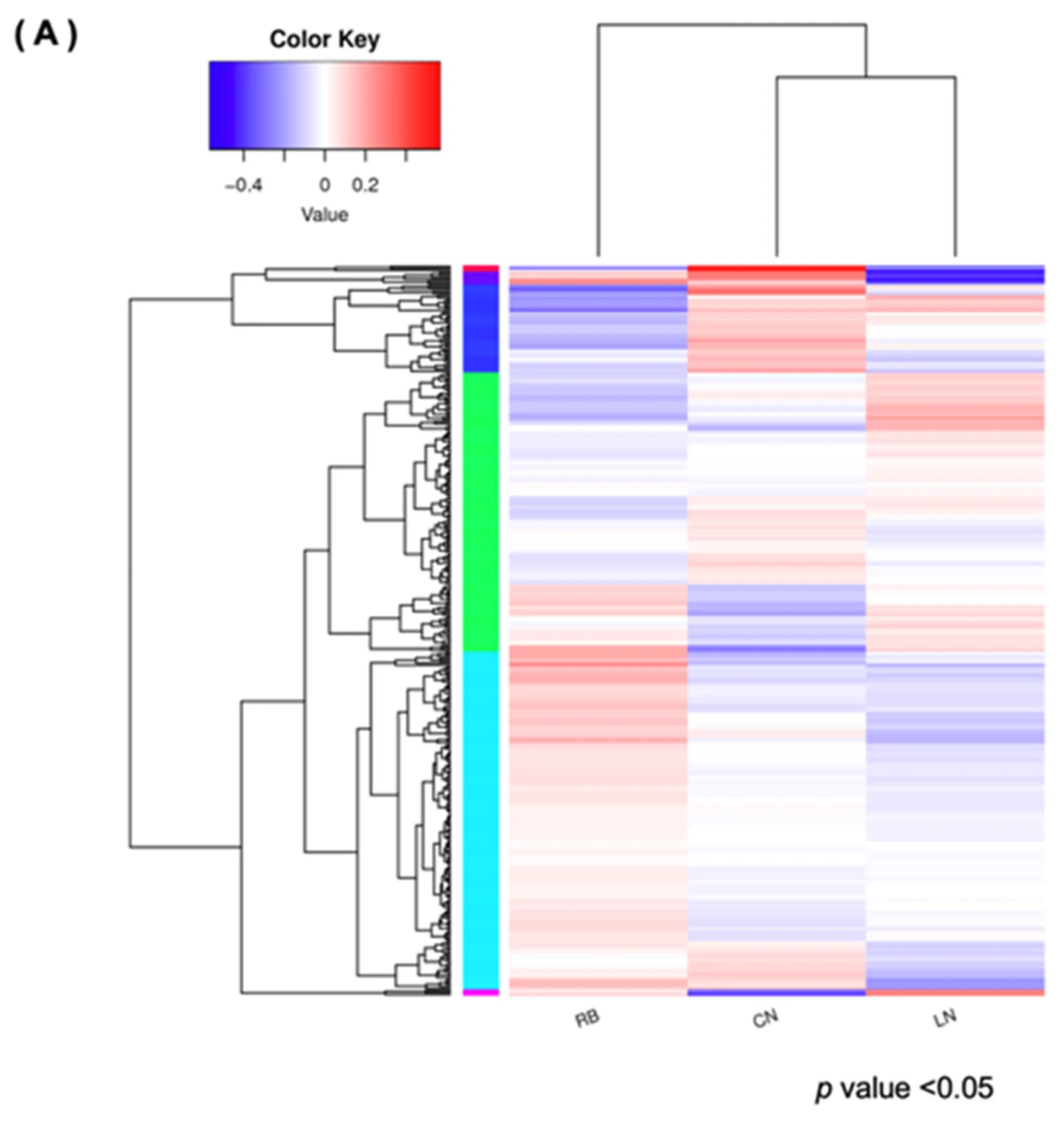

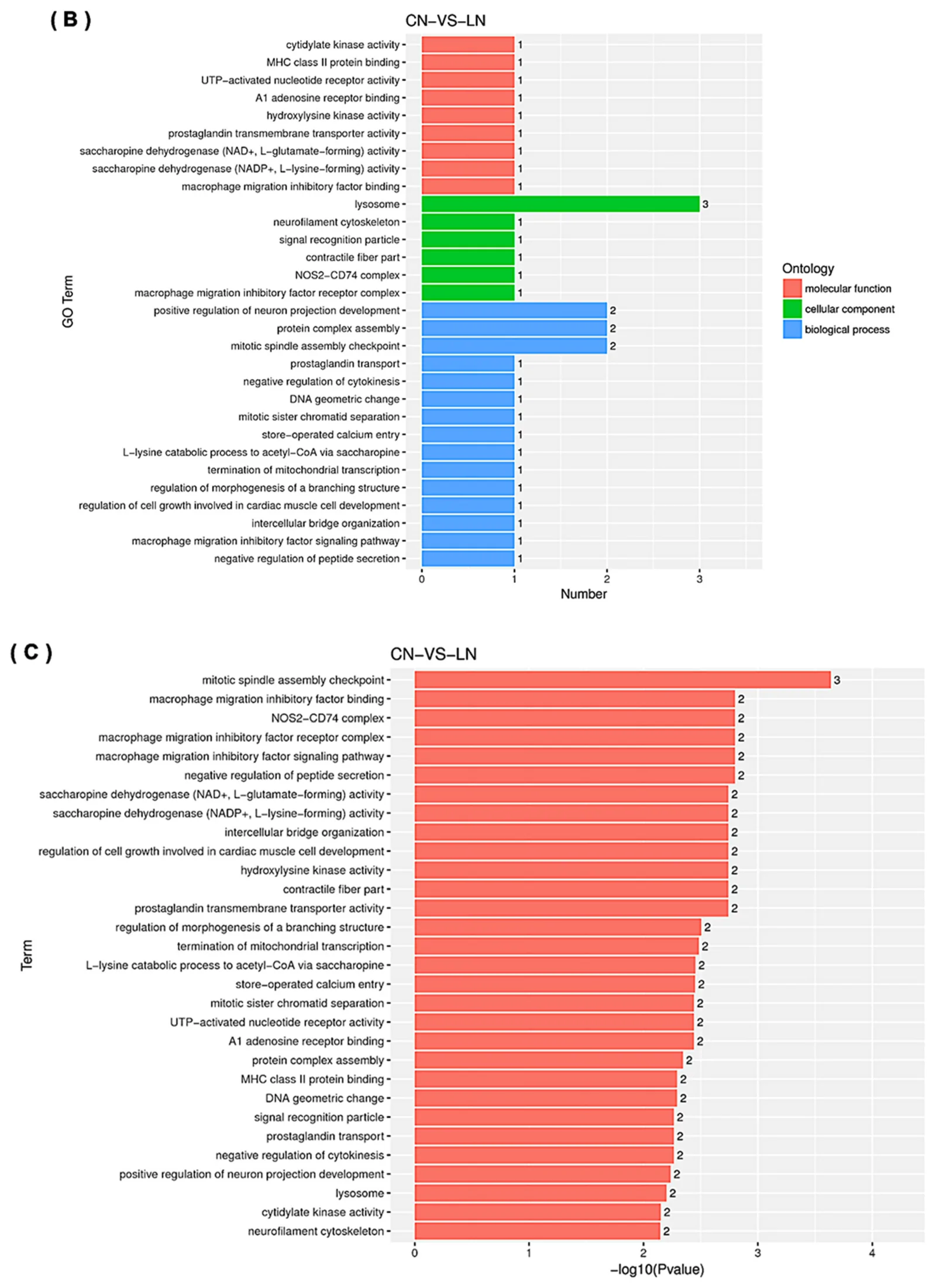
Hierarchical clustering and Gene Ontology (GO) functional enrichment analysis. (**A**) Hierarchical clustering heatmap showing global transcript profiles across all experimental groups (*p* < 0.05), with the color key indicating expression values. (**B**) GO enrichment analysis of candidate transcripts ranked by statistical significance (−log_10_ *p* value > 4) within the cellular component and biological process branches. (**C**) Enriched molecular function categories highlighting significantly altered networks in cell signaling, structural matrix dynamics, and immune modulation. Hierarchical clustering was performed using log_10_-transformed FPKM values, and functional enrichment was determined using the Wallenius non-central hypergeometric distribution model within the GOSeq pipeline.

To visualize the global shifting of these transcript profiles, we performed a hierarchical cluster analysis across all three experimental groups (Figure 3A, *p* < 0.05). The resulting heatmap reveals distinct blocks of co-regulated genes. To identify statistically overrepresented functional categories within the differentially expressed genes (DEGs), a Gene Ontology (GO) enrichment analysis was conducted and ranked by statistical significance ((−log_10_ *p*-value); Figure 3B,C). Within the cellular components branch, exploratory alterations were noted in transcripts localized to the lysosome (3 genes), providing preliminary observations regarding potential trafficking networks. In terms of biological processes, cell cycle regulation was the most heavily impacted category, with the mitotic spindle assembly checkpoint and sister chromatid separation emerging as the most statistically significant targets (*p* = 10^−4^), alongside positive regulation of neuron projection development and protein complex assembly (Figure 3B). Furthermore, molecular functions centered tightly on cell signaling, structural matrix dynamics, and immune modulation, showing significant enrichment in macrophage migration inhibitory factor (MIF) binding and receptor signaling, the NOS2–CD74 complex, MHC class II protein binding, saccharopine dehydrogenase activity (L-lysine catabolism), prostaglandin transmembrane transporter activity, and structural remodeling of the neurofilament cytoskeleton (Figure 3C).

### 2.4. Highly Upregulated and Downregulated Genes 

To characterize the transcriptomic alterations associated with linalool treatment in B16 cells, differential expression data were analyzed to identify prominent annotated candidate genes. The top 10 fully annotated upregulated and top 10 fully annotated downregulated genes are presented in Table 1, filtering out computationally predicted genes or uncharacterized loci without verified functional descriptions to ensure biological relevance. The broader 79 differentially regulated genes are detailed in Table S1. Linalool treatment was associated with distinct expression shifts in candidate networks involving cellular stress handling, vesicular dynamics, and mitotic progression. Notably, the treatment significantly upregulated key transcripts involved in intracellular transport and structural maintenance, including the actin-capping regulator *Avil* (log_2_ *FC* = 1.4), the lysosomal marker *Cd74* (log_2_ *FC* = 1.2), and the RAS oncogene family member *Rab25* (log_2_ *FC* = 3.6). 

Concurrently, macromolecular and metabolic transport cascades were stimulated, as evidenced by the upregulation of the prostaglandin transporter *Slco2a1* (log_2_ *FC* = 3.3) and the cytoplasmic kinase *Hykk* (log_2_ *FC* = 1.4), and the post-translational glycosyltransferase *Galnt6* (log_2_ *FC* = 3.6). Cell cycle machinery and alternative splicing pathways were also markedly shifted, demonstrated by the induction of *Tex14* (log_2_ *FC* = 3.5), *Khdc3* (log_2_ *FC* = 2.9), and structural U1 spliceosomal RNA (log_2_ *FC* = 4.2). 

Conversely, linalool treatment significantly downregulated several transcripts that modulate microenvironmental stress adaptation and cellular signaling. Specifically, transcripts vital for hypoxia adaptation, epigenetic regulation, and microenvironmetal responses were suppressed, such as the growth factor *Pgf* (log_2_ *FC* = −1.1), the pyroptosis responder *Gsdmd* (log_2_ *FC* = −1.0), the DNA methyltransferase Dnmt3c (log_2_ *FC* = −3.8), and the critical hypoxamir *Mir210* (log_2_ *FC* = −4.2). Mitochondrial homeostatic markers were also modulated, marked by the downregulation of the mitochondrial transcription termination factor *Mterf1b* (log_2_ *FC* = −1.1, *p* = 0.00225) and carboxylesterase *Ces1a* (log_2_ *FC* = −3.8). Furthermore, regulatory elements in secondary messenger systems, developmental pathways, and extracellular matrix remodeling networks were suppressed, highlighted by the downregulation of the calcium release-activated channel regulator *Cracr2a* (log_2_ *FC* = −1.1), the canonical Notch ligand *Dll1* (log_2_ *FC* = −3.8), the Wnt-signaling modulator *Cpz* (log_2_ *FC* = −1.5), *Col4a4* (log_2_ *FC* = −1.4), and *Adam11* (log_2_ *FC* = −1.3). Taken together, these transcriptomic patterns establish a comprehensive baseline of linalool-responsive expression shifts, identifying candidate pathways involved in cell cycle checkpoints, post-transcriptional organelle routing, and adaptive stress networks to guide future functional validation and therapeutic exploration in melanoma research.

### 2.5. Linalool’s Effect on Pigmentation-Associated Loci 

To evaluate the impact of linalool on the melanogenesis pathway, we analyzed the transcriptional behavior of classical coat-color genes. Differential expression analysis revealed that linalool treatment does not cause a profound global transcriptional repression of core melanogenic transcripts. Instead, it induces modest, nonsignificant transcriptional reduction in the master transcription factor *Mitf* (log_2_ fold change [*FC*] = −0.25) and its key downstream targets, including *Mc1r* (log_2_ *FC* = −0.18), *Mlana* (log_2_ *FC* = −0.12), *Dct* (log_2_ *FC* = −0.03), *Tyrp1* (log_2_ *FC* = −0.07), and the rate-limiting enzyme *Tyr* (log_2_ *FC* = −0.03). Intriguingly, despite these minimal mRNA-level shifts, parallel phenotypic validation via Western blot analysis confirmed a stark, significant reduction in functional tyrosinase (TYR) protein level. This distinct discrepancy indicates that pigment inhibition likely involves post-transcriptional, post-translational, or intracellular trafficking pathways, rather than a direct transcriptional blockade of the enzymatic loci. These observations align with the upregulation of *Rab25* (log_2_ *FC* = 1.6), a key transcript involved in vesicle recycling and endosomal–lysosomal transport, alongside a highly significant GO enrichment within the lysosomal cellular component category. 

### 2.6. Linalool Impairs Stress Adaptation and Redox Defense Networks 

Concurrently, differential expression profiling revealed that linalool (LN) treatment targeted candidate tracks within the melanoma cell expression architecture by significantly suppressing vital stress-adaptation and cellular plasticity regulators. This profile was highlighted by the profound down-regulation of microRNA 210 (*Mir210*, log_2_ *FC* = −4.2, *p* = 0.00316), a key non-coding RNA typically upregulated during hypoxia to maintain metabolic homeostasis and promote cell survival. In tandem, the cell-plasticity regulator delta-like canonical Notch ligand 1 (*Dll1*) was significantly suppressed, indicating an alteration in downstream candidate networks. This downregulation of adaptive regulators was consistent with parallel shifts in oxidative, metabolic, and xenobiotic detoxification networks across the dataset. Specifically, aminoadipate-semialdehyde synthase (*Aass*), an enzyme critically mapped to cellular oxidation-reduction processes (GO:0055114), was markedly repressed (log_2_ *FC* = −2.5), (*p* = 0.00124). Nuclear receptor subfamily 1 group I member 2 (*Nr1i2*), a major transcript driving xenobiotic metabolic processes (GO:0006805) and drug clearance (GO:0042738), was similarly downregulated (log_2_ *FC* = −2.4), (*p* = 0.00215), highlighting potential shifts in cellular detoxification pathways. Furthermore, placental growth factor (*Pgf*), which regulates key pathways involved in cellular responses to hypoxia (GO:0001666) and drug stress (GO:0042493), was concurrently down-regulated (log_2_ *FC* = −1.1, *p* = 0.00120), while gasdermin D (*Gsdmd*), involved in responding to extracellular stimuli (GO:0031668), was also suppressed (log_2_ *FC* = −1.0, *p* = 0.00218). Reflecting potential transcriptomic adaptations, the stress responder advillin (*Avil*, GO:0006950) was significantly upregulated (log_2_ *FC* = 1.4, *p* = 0.00055). 

## 3. Discussion

The skin acts as the body’s primary defense barrier against environmental insults, notably protecting against oxidative stress induced by harmful ultraviolet radiation (UVR) [22,23]. Melanin, the principal pigment in animal and human skin, hair, and eyes, is crucial for this defense. Melanin absorbs a broad UV-visible spectrum and efficiently dissipates up to 90% of absorbed solar energy as heat, an absorption capacity that is coupled with redox reactions and electron transfer processes within its polymer structure [24]. Mechanistically, melanins exist primarily as eumelanin (black–brown) and pheomelanin (red–yellow). Upon UV exposure, these pigments generate semiquinone-type free radical species. While eumelanin is relatively stable and large enough to effectively scavenge these reactive species, pheomelanin is photolabile, less efficient at radical scavenging, and its interaction with UVR can lead to a high production of reactive oxygen species (ROS), such as superoxide [25,26]. Consequently, a high pheomelanin content can sensitize cells to UV damage rather than provide robust protection [27].

This study establishes a comprehensive transcriptomic baseline profile of B16 melanoma cells treated with linalool, bridging phenotypic changes with global gene expression profiles. Phenotypic results demonstrated that linalool treatment across a concentration range of 0.1 to 100 µM does not induce cytotoxicity, with cell viability remaining near 100% and even showing a slight, statistically significant increase in proliferation (Figure 1A). Despite maintaining high cell viability, linalool significantly suppresses melanin production in a non-linear manner, where a 1 µM dose reduced melanin content to approximately 50% of control levels, exhibiting a stronger depigmenting effect than the standard positive control arbutin (~80%) (Figure 1B,D).

The distinct clustering observed in our PCA confirms that linalool induces a robust, reproducible shift in the transcriptomic landscape of B16 cells, separating clearly from both untreated controls and arbutin along PC2 (Figure 2B). This targeted global shift provides valuable candidate networks to complement our phenotypic findings, demonstrating that linalool suppresses melanin synthesis accompanied by a reduction in tyrosinase protein level without causing cytotoxicity (Figure 1). While arbutin typically reduces melanin synthesis by acting as a direct, competitive inhibitor of tyrosinase enzyme activity [28], our findings reveal that linalool employs a distinct, upstream mode of action centered on candidate structural and transcriptomic variations. 

**Figure 4:**
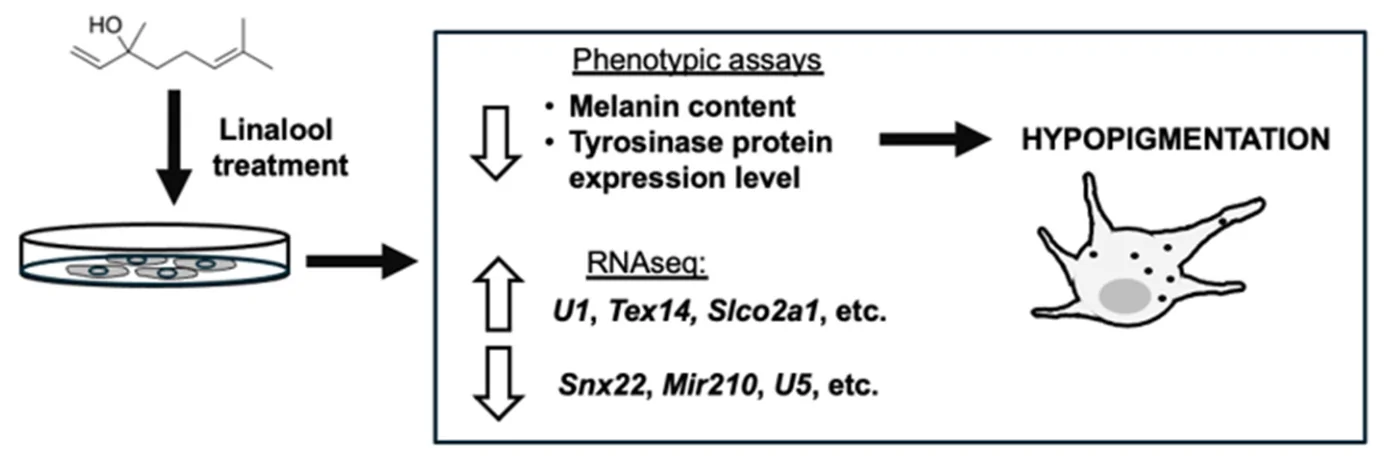
Hypothetical framework of linalool-mediated hypopigmentation and associated transcriptomic baseline shifts in B16 cells. Arrows denote directional trends (upregulation/downregulation).

The subsequent GO enrichment analysis connects these transcriptomic shifts directly to exploratory alterations in intracellular trafficking, cellular homeostasis, and immune-modulating pathways (Figure 3). Specifically, the localization of differentially expressed genes to the lysosome (3 genes) points to a targeted disruption of vesicular networks [29], as shown in Figure 3. Because melanosomes are specialized, lysosome-related organelles [30] responsible for melanin synthesis and transport [31], these transcriptomic shifts underscore how linalool targeted changes map onto these organelle dynamics (Figure 4). Candidate endosomal–lysosomal alterations align with the notable clearing of cell lysates (Figure 1C) and the ~50% reduction in pigment levels (Figure 1B) observed under linalool treatment. Furthermore, the profound enrichment in immune-modulating pathways [32,33,34] specifically macrophage migration inhibitory factor (MIF) binding, the NOS2–CD74 complex, and MHC class II protein binding (Figure 3), supports our screening focus on how linalool acts across multiple candidate pathways. Beyond its direct anti-melanogenic properties, highlighted by changes in the MIF–CD74 signaling axis which tumors routinely exploit to drive proliferation and evade host immunity [35,36], linalool concurrently exhibits transcriptional variations linked to checkpoint fidelity and structural remodeling of the neurofilament cytoskeleton (Figure 3). By altering these diverse signaling networks alongside structural remodeling, linalool provides a potent anti-melanogenic effect through a distinct transcriptomic baseline that maintains high cell viability, contrasting with conventional cytotoxic approaches. 

We analyzed the top differentially expressed transcripts (Table 1 and Table S1), and the screen successfully filters out computationally predicted loci, provisional non-coding elements, and uncharacterized RIKEN cDNA clones from the primary list of well-annotated, functional protein-coding effectors. These distinct expression profiles provide an objective baseline framework to explore candidate genetic networks regulating cell behavior [37]. Specifically, these annotated targets offer valuable insights into how this plant compound modulates melanogenesis and cellular oxidative stress pathways (the redox defense mechanism that cancer cells use to survive) [38]. Our transcriptomic findings demonstrate that linalool treatment suppresses key regulatory factors like *Mir210*, *Dll1*, and the oxidation-reduction enzyme *Aass* (Table 1). Rather than inducing active cytotoxicity at these doses, this targeted downregulation points to a latent state of molecular vulnerability [39,40], mapping potential regulatory tracks without reducing the immediate survival capacity of these melanoma cells. The coordinated transcriptional modulation of *Mir210*, *Dll1*, *Aass*, *Nr1i2*, *Pgf*, *Gsdmd*, and *Avil* indicates a responsive modification within redox-associated networks and cellular stress-adaptation systems. Together, these transcriptomic alterations suggest that linalool establishes a state of latent molecular vulnerability by modulating antioxidant defense signatures, mirroring the pathways previously documented in other cancer models [41,42]. 

Melanin synthesis is traditionally a tightly regulated process orchestrated by intricate intracellular signaling networks, primarily converging on the microphthalmia-associated transcription factor (MITF) [43]. The canonical pathway begins with the binding of alpha-melanocyte stimulating hormone (α-MSH) to the melanocortin 1 receptor (MC1R) on the melanocyte surface. This G-protein coupled receptor activation increases intracellular cyclic adenosine monophosphate (cAMP) levels, which in turn activates protein kinase A (PKA). PKA then phosphorylates the cAMP-response element-binding protein (CREB), which translocates to the nucleus and enhances the transcription of the MITF gene. MITF acts as the master regulator, upregulating the expression and activity of key melanogenic enzymes, including tyrosinase (TYR), tyrosinase-related protein 1 (TRP-1), and TRP-2, thereby promoting melanin production [44,45].

Intriguingly, while Western blot analysis in this study demonstrated that linalool treatment markedly decreased active TYR protein expression levels (Figure 1D), our global transcriptomic screening revealed that the classical upstream components of this pathway, including *Mitf* (log_2_ *FC* = −0.25), *Mc1r* (log_2_ *FC* = −0.18), and key downstream enzymes Tyr (log_2_ *FC* = −0.03), *Tyrp1* (log_2_ *FC* = −0.07), and *Dct*/*Tyrp2* (log_2_ *FC* = −0.03), exhibited only minor, non-significant transcriptional down-regulation (Table 1). This sharp discrepancy between minimal mRNA-level shifts and a stark reduction in TYR protein indicates that linalool bypass direct transcriptional blockade of the canonical pathway. Instead, the sharp induction of functional genes involved in macromolecular movement and vesicle recycling, such as *Rab25* (Table S1), suggests that linalool is associated with distinct variations in internal intracellular trafficking networks (Figure 3A,B) [46]. As *Rab25* is a key effector of membrane transport [47], its upregulation aligns with an exploratory reorganization or modification of the specialized endosomal–lysosomal vesicles (melanosomes) responsible for pigment maturation and transport [48]. Rather than silencing the genes at the nuclear level, these transcriptomic patterns highlight candidate networks governing vesicle trafficking, post-transcriptional sorting, and structural components of the pigment-producing machinery, providing baseline insights into altered tyrosinase distribution across lysosomal structures (Figure 3A,B).

Our analysis of the pigmentation loci suggests that linalool modulates phenotypic pigmentation, aligning with exploratory shifts in the endosomal–lysosomal and vesicle trafficking networks associated with melanosome assembly, maturation, and intracellular transport. In contrast to linalool’s non-cytotoxic regulatory profile, other natural products like daphnane diterpene hirsein A and B have been shown to suppress melanogenesis by downregulating the expression of the transcription factor *Mitf* to inhibit pigmentation and melanosome transport in pigment cell models [49,50]. Consequently, to expand upon this baseline for potential broader applications, it is valuable to evaluate linalool’s specific regulatory networks in healthy, non-malignant melanocytes.

In conclusion, this study provides a comprehensive transcriptomic characterization of the hypopigmentation networks induced by the plant-derived phytochemical linalool in B16 cells. Phenotypically, this natural bioactive suppresses melanin synthesis and limits functional tyrosinase protein abundance, displaying an optimal non-linear efficacy at low micromolar concentrations. Structurally, our RNA-seq and GO analyses reveal that this pigmentation suppression does not rely on direct global transcriptional repression of core coat-color loci (*Mitf*, *Tyr*, *Dct*); rather, it highlights candidate regulatory networks involving endosomal–lysosomal and vesicle trafficking systems, with transport effectors like *Rab25* serving as prominent targets. Simultaneously, linalool downregulates vital stress-adaptation, cell-plasticity, and cellular detoxification regulators (*Mir210*, *Dll1*, *Aass*, *Nr1i2*, *Pgf*), pointing to a state of latent molecular vulnerability that mirrors the oxidative responses reported in colon cancer cells. While this system provided a useful baseline for mapping these networks, a limitation of the current study is its reliance on a single murine cell line model. To build upon these findings, future studies utilizing targeted genetic knockdowns and quantitative functional assays are required to definitively corroborate these specific transcriptomic networks. Furthermore, evaluating the non-malignant cellular counterpart of B16 cells, such as normal murine or human melanocytes, would provide valuable insights into the selectivity and safety profile of linalool. Overall, this study provides critical insights establishing a robust mechanistic foundation and uncovering highly specific molecular targets to guide future functional validation, thereby accelerating the translational development and therapeutic exploration of this botanical nutraceutical agent in melanoma and hyperpigmentation research.

## 4. Materials and Methods

### 4.1. Cell Culture 

B16 murine melanoma cells (B16-F10 cells; Cell No. RCB2630) were purchased from the Riken Cell Bank in Tsukuba City, Japan, and maintained as a monolayer culture in Dulbecco’s modified eagle medium (DMEM; Sigma, St Louis, MO, USA), supplemented with 10% fetal bovine serum or FBS (Sigma), 50 U⁄mL penicillin and 50 µg/mL streptomycin (Cambrex, East Rutherford, NJ, USA). The cells were subcultured in 75 cm^2^ flasks (Corning, Inc, New York, NY, USA) and incubated at 37 °C in a 5% CO_2_-humidified incubator. The cell culture medium was changed twice a week, and the cells were subcultured upon reaching 80% confluence. For treatments, stock solution of racemic (±)-linalool (97% purity, L2602; Sigma-Aldrich, St. Louis, MO, USA) was dissolved in 70% ethanol (WAKO, Osaka, Japan) and diluted into the culture medium immediately before use. This vehicle was strictly included in all negative control groups at an identical final concentration. Stock solutions were stored fresh in airtight, light-protected containers under refrigerated conditions to limit volatilization. Arbutin (4-hydroxyphenyl-beta-D-glucopyranoside; 8% purity; Sigma-Aldrich, St. Louis, MO, USA) was selected as a positive reference control based on established literature parameters for benchmarking tyrosinase inhibition in B16 cells.

### 4.2. Cell Proliferation Assay

The effect of linalool on B16 cell proliferation was evaluated using the 3-(4,5-dimethylthiazol-2-yl)-2,5-diphenyltetrazolium bromide (MTT; Sigma-Aldrich, St. Louis, MO, USA) assay [51]. B16 cells were seeded onto 96-well plates at a density of 3 × 10^3^ cells/well and incubated for 24 h. The culture medium was then replaced with fresh medium containing various concentrations of linalool (0, 0.1, 1, 10, 100 μM) or arbutin (100 μM). After 24 h incubation at 37 °C in a humidified atmosphere of 95% air and 5% CO_2_, the MTT reagent (5 mg/mL) was added to each well at a volume of 10 μL/well (0.45 mg/mL final concentration). The plates were then incubated at 37 °C for 4 h in the dark by wrapping them in aluminum foil. Sodium dodecyl sulfate (SDS; 10%; Fujifilm WAKO Pure Chemical Corporation, Osaka, Japan) was then added to each well, followed by overnight incubation at 37 °C to completely dissolve the formazan crystals. Absorbance values were measured at 570 nm using a microplate reader (Powerscan HT; Dainippon Pharmaceuticals USA Corporation, Osaka, Japan). Blanks containing only the culture medium, MTT, and SDS were utilized to correct the optical density values.

### 4.3. Melanin Assay 

The melanin content of the cells was determined following the protocol described previously [52]. B16 cells were cultured at a density of 5 × 10^5^ cells in 100-mm Petri dishes and incubated as described above. After overnight incubation, the culture medium was replaced with fresh sample-containing culture medium and incubated further. After 48 h incubation, the culture medium was removed, and the cells were washed twice with phosphate-buffered saline (PBS). The cells were then detached by adding trypsin (0.25% trypsin/0.02% EDTA in PBS; Sigma-Aldrich, St. Louis, MO, USA) to collect them. The cells were then pelleted by centrifugation and solubilized by adding 0.1% Triton X-100 (Sigma-Aldrich, Burlington, MA, USA), after which the melanin was precipitated by adding 10% trichloroacetic acid (Sigma-Aldrich, Burlington, MA, USA). The isolated melanin was then dissolved in 8 N NaOH (Fujifilm WAKO Pure Chemical Corp., Osaka, Japan) and incubated for 2 h at 80 °C. The amount of melanin in the solution was determined spectrophotometrically by measuring the absorbance at 410 nm. The total melanin content was estimated using a standard curve generated with synthetic melanin (M0418; Sigma-Aldrich, Burlington, MA, USA) and was expressed as melanin content per viable cell. The number of viable cells was determined flow cytometrically using the ViaCount program of a Guava PCA platform (GE Healthcare UK Ltd., Buckinghamshire, UK) following the manufacturer’s instructions.

### 4.4. Western Blot Analysis 

B16 melanoma cells were seeded onto 100 mm dishes at a density of 3 × 10^6^ cells/dish and incubated as described above. After overnight incubation, the medium was replaced with sample-containing medium, followed by incubation for 48 h. The medium was then removed, the cells were washed twice with PBS, and total protein extraction was carried out using RIPA lysis buffer (Sigma-Aldrich, Burlington, MA, USA) following the manufacturer’s instructions. SDS–polyacrylamide gel electrophoresis (10% SDS-PAGE) was performed to resolve 15 µg of extracted protein sample per lane, after which the proteins were blotted onto a nitrocellulose membrane. To detect tyrosinase, the membrane was incubated in blocking buffer (LI-COR Biosciences, Lincoln, NE, USA) followed by incubation with primary anti-tyrosinase polyclonal antibody (Santa Cruz Biotechnology, Inc., Santa Cruz, CA, USA; 1:200 dilution). After incubation with HRP-labeled secondary antibody (Santa Cruz Biotechnology, Inc., Dallas, TX, USA), the chemiluminescent signal was visualized using Immobilon Western Chemiluminescent HRP Substrate (Millipore, Burlington, MA, USA). The densitometric intensities of the protein bands were subsequently quantified using ImageJ 1.54g software (National Institutes of Health, Bethesda, MD, USA).

### 4.5. RNA Extraction and Quantification 

Total RNA was isolated using the ISOGEN kit (Nippon Gene, Tokyo, Japan) according to the manufacturer’s protocols. Due to initial spectrophotometric assessments indicating potential salt or solvent carryover (A_260_/A_230_ ratios of 0.69–0.89), the crude RNA extracts were subjected to an additional cleanup step using an RNA purification column to remove residual contaminants. The final quality and concentration of the purified RNA were determined using a NanoDrop 2000 spectrophotometer (Thermo Fisher Scientific, Wilmington, DE, USA), yielding optimized A_260_/A_280_ ratios of 1.98–2.01. The structural integrity of the total RNA was further validated via agarose gel electrophoresis, confirming sharp, intact ribosomal bands with no visible signs of degradation.

### 4.6. RNAseq Analyses 

Strand-specific RNA-sequencing was performed by Azenta Life Sciences (Genewiz; Tokyo, Japan). Polyadenylated RNA transcripts were isolated from 1 μg of high-quality total RNA using Oligo(dT) magnetic beads for poly-A selection library preparation. The enriched mRNA samples were fragmented and reverse-transcribed using random primers to construct indexed libraries, which were multiplexed and sequenced on an Illumina platform using a 2 × 150 bp paired-end (PE) configuration. Raw sequencing reads were processed using Cutadapt (v1.9.1) to trim adapter sequences and filter out low-quality bases (Phred quality score *Q* < 20). The clean, high-quality reads were aligned to the mouse reference genome using Hisat2 (v2.0.1). Gene expression levels were quantified using HTSeq (v0.6.1)**,** and differential expression analysis was performed using the negative binomial distribution model within the DESeq2 Bioconductor package to calculate adjusted *p*-values (*p*_adj_) controlling the false discovery rate via the Benjamini–Hochberg procedure. Functional annotation and Gene Ontology (GO) terms were analyzed using the Wallenius non-central hypergeometric distribution model within the GOSeq (v1.34.1) and topGO (v2.22.0) software packages.

### 4.7. Statistical Evaluation 

The normality of the data distribution was evaluated prior to statistical analysis. Data from phenotypic assays (cell proliferation and melanin content quantification) are expressed as the mean ± standard error of the mean (SEM). For multiple-dose comparisons, statistical significance was determined using a one-way Analysis of Variance (ANOVA) followed by Dunnett’s post hoc test to compare each linalool treatment group against the untreated control, with independently harvested wells serving as the experimental unit across independent biological replicates (*n* = 3). For high-throughput transcriptomic screening and functional annotation data, statistical thresholds and false discovery rates were calculated using the Wald test with Benjamini–Hochberg adjustments (*p*_adj_ < 0.05) within the DESeq2 pipeline, while Gene Ontology enrichment significance was determined via the Wallenius non-central hypergeometric distribution model within the GOSeq software package.

## Figures and Tables

**Table 1:** Top 10 upregulated and top 10 downregulated fully annotated differentially expressed genes in linalool-treated B16 melanoma cells *.

Gene Symbol	Description	log_2_ Fold Change	*p* Value	Q Value (*p_adj_*)
*U1*	U1 spliceosomal RNA	4.2	0.0027	0.0411
*Tex14*	testis-expressed gene 14	3.5	0.0018	0.0311
*Slco2a1*	solute carrier organic anion transporter family member 2a1	3.3	0.0034	0.0490
*Rps26-ps1*	ribosomal protein S26 pseudogene 1	3.0	0.0000	0.0000
*Khdc3*	KH domain containing 3 subcortical maternal complex members	2.9	0.0007	0.0159
*Elf5*	E74-like factor 5	2.8	0.0013	0.0254
*Shd*	src homology 2 domain-containing transforming protein D	2.8	0.0013	0.0254
*Dusp23*	Dual-specificity phosphatase 23	2.1	0.0028	0.0424
*Gm22362*	predicted gene 22362	2.1	0.0028	0.0424
*Synm*	synemin intermediate filament protein	1.9	0.0005	0.0121
*Mblac1*	metallo-beta-lactamase domain containing 1	−1.8	0.0021	0.0350
*Fam177a2*	family with sequence similarity 177 member A2	−2.1	0.0000	0.0000
*Nr1i2*	nuclear receptor subfamily 1 group I member 2	−2.4	0.0021	0.0352
*Aass*	aminoadipate-semialdehyde synthase	−2.5	0.0012	0.0238
*Irs3*	insulin receptor substrate 3	−2.7	0.0032	0.0465
*P2ry2*	purinergic receptor P2Y2 G-protein coupled 2	−2.8	0.0000	0.0005
*Col14a1*	collagen-type XIValpha 1	−3.2	0.0001	0.0031
*Snx22*	sorting nexin 22	−3.5	0.0022	0.0363
*Mir210*	microRNA 210	−4.2	0.0032	0.0462
*U5*	U5 spliceosomal RNA	−4.2	0.0032	0.0462

* Differentially expressed genes (DEGs) in B16 melanoma cells following linalool treatment versus untreated control. Statistical boundaries were calculated using the standard DESeq2 negative binomial Wald testing framework. Multiple-testing corrections were strictly applied using the Benjamini–Hochberg false discovery rate (FDR) method to generate adjusted *p*-values (nominal and adjusted, *p_adj_*). All unannotated predicted loci and pseudogenes were excluded from this index.

## Data Availability

The original data presented in the study are available upon request to the corresponding authors.

## Supplementary Materials

The following supporting information can be downloaded at: https://www.mdpi.com/article/10.3390/ijms27208956/s1.

## Abbreviations

The following abbreviations are used in this manuscript:

MTT	3-(4,5-dimethylthiazol-2-yl)-2,5-diphenyl-tetrazolium bromide
SDS-PAGE	sodium dodecyl sulfate–polyacrylamide gel electrophoresis
GAPDH	Glyceraldehyde 3-phosphate dehydrogenase
RNA-seq	RNA sequencing
PCA	Principal component analysis
GO	Gene ontology
MITF	microphthalmia-associated transcription factor
TYR	tyrosinase
*Mir210*	microRNA-210
*Rab25*	RAB25, member RAS oncogene family

## References

[1] Diniz do Nascimento L., Barbosa de Moraes A.A., Santana da Costa K., Pereira Galúcio J.M., Taube P.S., Leal Costa C.M., Neves Cruz J., de Aguilar Andrade E.H., Guerreiro de Faria L.J. (2020). Bioactive natural compounds and antioxidant activity of essential oils from spice plants: New findings and potential applications. Biomolecules.

[2] Villareal M.O., Mopera L.E., Completo G.C., Nacario R.C., Romero M.V., Castillo-Israel K.A.T., Lizardo-Agustin R.C.M., Laya M.J.G., Obdosantos R.M.M., Romano C.L.O. (2025). Philippine functional foods: A new frontier in noncommunicable diseases prevention. Food Humanit..

[3] An Q., Ren J.N., Li X., Fan G., Qu S.S., Song Y., Li Y., Pan S.Y. (2021). Recent updates on bioactive properties of linalool. Food Funct..

[4] Aprosoaoie A.C., Hâncianu M., Costache I.I., Miron A. (2014). Linalool: A review on a key odorant molecule with valuable biological properties. Flavour Fragr. J..

[5] Özek G., Schepetkin I.A., Yermagambetova M., Özek T., Kirpotina L.N., Almerekova S.S., Abugalieva S.I., Khlebnikov A.I., Quinn M.T. (2021). Innate immunomodulatory activity of cedrol, a component of essential oils isolated from *Juniperus* species. Molecules.

[6] Maurya R., Gupta P., Chanotiya C.S., Dhawan S.S., Srivastava S.S., Yadav A., Kumar A., Swamy Y., Lal R.K. (2022). Investigation of monoterpenoids rich essential oils of two *Ocimum basilicum* L. varieties at different agro-climatic conditions in India. Acta Ecol. Sin..

[7] Guo F., Chen Q., Liang Q., Zhang M., Chen W., Chen H., Yun Y., Zhong Q., Chen W. (2021). Antimicrobial Activity and Proposed Action Mechanism of Linalool Against Pseudomonas fluorescens. Front. Microbiol..

[8] Bickers D., Calow P., Greim H., Hanifin J.M., Rogers A.E., Saurat J.H., Sipes I.G., Smith R.L., Tagami H. (2003). A toxicologic and dermatologic assessment of linalool and esters when used as fragrance ingredients. Food Chem. Toxicol..

[9] Bar S., Kara M. (2024). Linalool exerts antioxidant activity in a rat model of diabetes by increasing catalase activity without antihyperglycemic effect. Exp. Ther. Med..

[10] Rojas-Armas J.P., Arroyo-Acevedo J.L., Palomino-Pacheco M., Ortiz-Sánchez J.M., Justil-Guerrero H.J., Martínez-Heredia J.T., Dongo R.J.Z. (2025). Pharmacological and molecular insights into linalool-rich *Coriandrum sativum* essential oil: Anticonvulsant, analgesic, anti-inflammatory, and antioxidant potential in rodent models. Vet. World.

[11] Souto-Maior F.N., Fonsêca D.V.D., Salgado P.R.R., Monte L.D.O., de Sousa D.P., de Almeida R.N. (2017). Antinociceptive and anticonvulsant effects of the monoterpene linalool oxide. Pharm. Biol..

[12] Seol G.H., Kang P., Lee H.S., Seol G.H. (2016). Antioxidant activity of linalool in patients with carpal tunnel syndrome. BMC Neurol..

[13] Park H., Seol G.H., Ryu S., Choi I.Y. (2016). Neuroprotective effects of (−)-linalool against oxygen-glucose deprivation-induced neuronal injury. Arch. Pharmacal Res..

[14] Gunaseelan S., Balupillai A., Govindasamy K., Ramasamy K., Muthusamy G., Shanmugam M., Thangaiyan R., Robert B.N., Nagarajan R.P., Ponniresan V.K. (2017). Linalool prevents oxidative stress activated protein kinases in single UVB-exposed human skin cells. PLoS ONE.

[15] Iwasaki K., Zheng Y.W., Murata S., Ito H., Nakayama K., Kurokawa T., Sano N., Nowatari T., Villareal M.O., Nagano Y.N. (2016). Anticancer effect of linalool via cancer-specific hydroxyl radical generation in human colon cancer. World J. Gastroenterol..

[16] Rodenak-Kladniew B., Castro M.A., Crespo R., Galle M., de Bravo M.G. (2020). Anti-cancer mechanisms of linalool and 1, 8-cineole in non-small cell lung cancer A549 cells. Heliyon.

[17] Ito S., Sugumaran M., Wakamatsu K. (2020). Chemical reactivities of *ortho*-quinones produced in living organisms: Fate of quinonoid products formed by tyrosinase and phenoloxidase action on phenols and catechols. Int. J. Mol. Sci..

[18] Jenkins N.C., Grossman D. (2013). Role of melanin in melanocyte dysregulation of reactive oxygen species. BioMed Res. Int..

[19] Kamiński K., Kazimierczak U., Kolenda T. (2022). Oxidative stress in melanogenesis and melanoma development. Contemp. Oncol./Współczesna Onkol..

[20] Andrei F., Ersilia A., Tulcan C., Dragomirescu A. (2018). Chemical composition and the potential of *Lavandula angustifolia* L. oil as a skin depigmentant. Rec. Nat. Prod..

[21] Ramsden C.A., Riley P.A. (2014). Tyrosinase: The four oxidation states of the active site and their relevance to enzymatic activation, oxidation and inactivation. Bioorg. Med. Chem..

[22] Papaccio F., Caputo S., Bellei B. (2022). Focus on the contribution of oxidative stress in skin aging. Antioxidants.

[23] Mohania D., Chandel S., Kumar P., Verma V., Digvijay K., Tripathi D., Choudhury K., Mitten S.K., Shah D. (2017). Ultraviolet radiations: Skin defense-damage mechanism. Ultraviolet Light in Human Health, Diseases and Environment.

[24] Mostert A.B. (2021). Melanin, the what, the why and the how: An introductory review for materials scientists interested in flexible and versatile polymers. Polymers.

[25] Solano F. (2020). Photoprotection and skin pigmentation: Melanin-related molecules and some other new agents obtained from natural sources. Molecules.

[26] Herrling T., Jung K., Fuchs J. (2008). The role of melanin as protector against free radicals in skin and its role as free radical indicator in hair. Spectrochim. Acta Part A Mol. Biomol. Spectrosc..

[27] Ito S., Wakamatsu K., Sarna T. (2018). Photodegradation of eumelanin and pheomelanin and its pathophysiological implications. Photochem. Photobiol..

[28] Saeedi M., Khezri K., Seyed Zakaryaei A., Mohammadamini H. (2021). A comprehensive review of the therapeutic potential of α-arbutin. Phytother. Res..

[29] Nturubika B.D., Guardia C.M., Gershlick D.C., Logan J.M., Martini C., Heatlie J.K., Lazniewska J., Moore C., Lam G.T., Li K.L. (2024). Altered expression of vesicular trafficking machinery in prostate cancer affects lysosomal dynamics and provides insight into the underlying biology and disease progression. Br. J. Cancer.

[30] Barral D.C., Delevoye C., Larue L., Seabra M.C., Raposo G., Setty S.R.G. (2025). Insights into lysosome-related organelle biogenesis: Melanosome as a model organelle. Front. Cell Dev. Biol..

[31] Bao M., Gempeler M., Campiche R. (2025). Melanosome transport and processing in skin pigmentation: Mechanisms and targets for pigmentation modulation. Int. J. Mol. Sci..

[32] Roger T., Schneider A., Weier M., Sweep F.C., Le Roy D., Bernhagen J., Calandra T., Giannoni E. (2016). High expression levels of macrophage migration inhibitory factor sustain the innate immune responses of neonates. Proc. Natl. Acad. Sci. USA.

[33] Tanese K., Hashimoto Y., Berkova Z., Wang Y., Samaniego F., Lee J.E., Ekmekcioglu S., Grimm E.A. (2015). Cell surface CD74–MIF interactions drive melanoma survival in response to interferon-γ. J. Investig. Dermatol..

[34] Axelrod M.L., Cook R.S., Johnson D.B., Balko J.M. (2019). Biological Consequences of Major Histocompatibility Class-II Expression by Tumor Cells in Cancer. Clin. Cancer Res..

[35] Fey R.M., Nichols R.A., Tran T.T., Vandenbark A.A., Kulkarni R.P. (2024). MIF and CD74 as emerging biomarkers for immune checkpoint blockade therapy. Cancers.

[36] Yan L., Wu M., Wang T., Yuan H., Zhang X., Zhang H., Li T., Pandey V., Han X., Lobie P.E. (2024). Breast cancer stem cells secrete MIF to mediate tumor metabolic reprogramming that drives immune evasion. Cancer Res..

[37] Schadendorf D., Van Akkooi A.C., Berking C., Griewank K.G., Gutzmer R., Hauschild A., Stang A., Roesch A., Ugurel S. (2018). Melanoma. Lancet.

[38] Bonaventure J., Domingues M.J., Larue L. (2013). Cellular and molecular mechanisms controlling the migration of melanocytes and melanoma cells. Pigment. Cell Melanoma Res..

[39] Abolhasani S., Ahmadi Y., Fattahi D., Rostami Y., Chollou K.M. (2025). MicroRNA-Mediated Regulation of Oxidative Stress in Cardiovascular Diseases. J. Clin. Lab. Anal..

[40] Chen L., Lu H., Peng D., Cao L., Chen Z., Bhat A., Wang T.C., Capobianco A., Que J., McDonald O.G. (2022). Activation of NOTCH signaling via DLL1 is mediated by APE1-redox-dependent NF-kB activation in esophageal adenocarcinoma. Cancer Res..

[41] Totten S.P., Im Y.K., Cepeda Cañedo E., Najyb O., Nguyen A., Hébert S., Ahn R., Lewis K., Lebeau B., Le Selba R. (2021). STAT1 potentiates oxidative stress revealing a targetable vulnerability that increases phenformin efficacy in breast cancer. Nat. Commun..

[42] Hileman E.O., Liu J., Albitar M., Keating M.J., Huang P. (2004). Intrinsic oxidative stress in cancer cells: A biochemical basis for therapeutic selectivity. Cancer Chemother. Pharmacol..

[43] Goding C.R., Arnheiter H. (2019). MITF—The first 25 years. Genes Dev..

[44] D’Mello S.A., Finlay G.J., Baguley B.C., Askarian-Amiri M.E. (2016). Signaling pathways in melanogenesis. Int. J. Mol. Sci..

[45] Byun K. (2025). Melanogenesis Pathway in the Skin. Melanin: A Biological Perspective and Aesthetic Approach.

[46] Stenmark H. (2009). Rab GTPases as coordinators of vesicle traffic. Nat. Rev. Mol. Cell Biol..

[47] Jin H., Tang Y., Yang L., Peng X., Li B., Fan Q., Wei S., Yang S., Li X., Wu B. (2021). Rab GTPases: Central coordinators of membrane trafficking in cancer. Front. Cell Dev. Biol..

[48] Goldenring J.R. (2013). A central role for vesicle trafficking in epithelial neoplasia: Intracellular highways to carcinogenesis. Nat. Rev. Cancer.

[49] Villareal M.O., Han J., Ikuta K., Isoda H. (2011). Daphnane diterpene hirsein B downregulates melanogenesis in B16 murine melanoma cells by cAMP pathway inhibition. BMC Proc..

[50] Villareal M.O., Han J., Yamada P., Shigemori H., Isoda H. (2010). Hirseins inhibit melanogenesis by regulating the gene expressions of *Mitf* and melanogenesis enzymes. Exp. Dermatol..

[51] Mosmann T. (1983). Rapid colorimetric assay for cellular growth and survival: Application to proliferation and cytotoxicity assays. J. Immunol. Methods.

[52] Villareal M.O., Chaochaiphat T., Bejaoui M., Sato K., Isoda H. (2020). Tara tannin regulates pigmentation by modulating melanogenesis enzymes and melanosome transport proteins expression. Planta Medica Int. Open.

