# Supplementary material for: Transcriptome Analysis of Linalool-Induced Hypopigmentation in B16 Melanoma Cells
Source: Int J Mol Sci. 2026 Oct 9;27(20):8956. doi: 10.3390/ijms27208956 (PMC13704213; doi:10.3390/ijms27208956)
Supplement: Supplementary file 1 [file ijms-27-08956-s001.zip › ijms-4601699-supplementary.pdf]

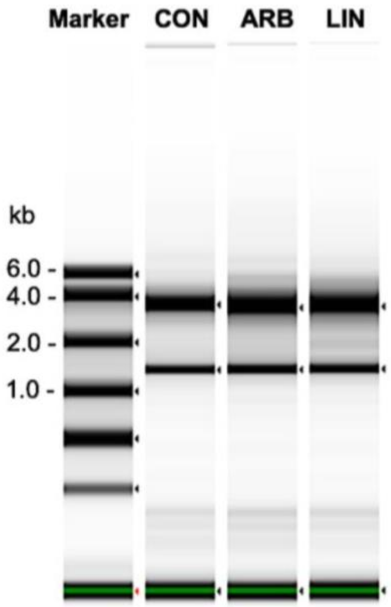

Figure S1. Gel electrophoresis analysis of total RNA samples extracted from treated experimental groups. The lane labeled Marker indicates the RNA size standard in kilobases (kb). Lanes CON, ARB, and LIN represent the total RNA extracted from the control, arbutin, and linalool treatment conditions, respectively. The prominent bands visible between 2.0 kb and 4.0 kb, as well as around 1.0 kb to 2.0 kb, correspond to the intact ribosomal RNA (rRNA) subunits (28S and 18S), demonstrating high quality and integrity across all experimental samples.

Table S1. List of candidate differentially expressed genes (DEGs) and their associated Gene Ontology terms in linalool-treated B16 cells compared to the untreated controls*

Gene Symbol	Description	log_2 Fold Change	p value	P adj	Gene Ontology		
					Biological Process	Cellular Component	Molecular Function
U1	U1 spliceosomal RNA	4.2	0.0027	0.0411	-	-	-
Tex14	testis expressed gene 14	3.5	0.0018	0.0311	GO:0032091:negative regulation of protein binding;GO:0008608:attachment of spindle microtubules to kinetochore;GO:0043063:intercellular bridge organization;GO:0006468:protein phosphorylation;GO:0007140:male meiosis;GO:0032466:negative regulation of cytokinesis;GO:0007094:mitotic spindle assembly checkpoint;GO:0051306:mitotic sister chromatid separation;	GO:0005737:cytoplasm;GO:0000776:kinetochore;GO:0045171:intercellular bridge;GO:0030496:midbody;	GO:0019901:protein kinase binding;GO:0004672:protein kinase activity;GO:0005524:ATP binding;
Slco2a1	solute carrier organic anion transporter family member 2a1	3.3	0.0034	0.0490	GO:0015732:prostaglandin transport;GO:0055085:transmembrane transport;	GO:0005887:integral component of plasma membrane;	GO:0015132:prostaglandin transmembrane transporter activity;
Khdc3	KH domain containing 3 subcortical maternal complex member	2.9	0.0007	0.0159	GO:0006468:protein phosphorylation;GO:0007094:mitotic spindle assembly checkpoint;GO:0090307:mitotic spindle assembly;GO:0007275:multicellular organismal development;	GO:0043234:protein complex;GO:0045179:apical cortex;	GO:0003723:RNA binding;GO:0005515:protein binding;

Elf5	E74-like factor 5	2.8	0.0013	0.0254	GO:0006355:regulation of transcription, DNA-templated;GO:0060644:mammary gland epithelial cell differentiation;GO:0007398:ectoderm development;	GO:0005737:cytoplasm;GO:0005634:nucleus;	GO:0043565:sequence-specific DNA binding;GO:0003700:sequence-specific DNA binding transcription factor activity;
Shd	src homology 2 domain-containing transforming protein D	2.8	0.0013	0.0254	-	-	-
Dusp23	dual specificity phosphatase 23	2.1	0.0028	0.0424	GO:0006470:protein dephosphorylation;	GO:0005737:cytoplasm;GO:0005634:nucleus;	GO:0004725:protein tyrosine phosphatase activity;GO:0008138:protein tyrosine/serine/threonine phosphatase activity;
Synm	synemin%2C intermediate filament protein	1.9	0.0005	0.0121	-	GO:0030018:Z disc;GO:0005912:adherens junction;GO:0043034:costamere;GO:0005882:intermediate filament;GO:0060053:neurofilament cytoskeleton;GO:0016020:membrane;GO:0030054:cell junction;GO:0044449:contractile fiber part;GO:0030016:myofibril;	GO:0005200:structural constituent of cytoskeleton;GO:0008307:structural constituent of muscle;GO:0030674:protein binding, bridging;GO:0017166:vinculin binding;GO:0019215:intermediate filament binding;GO:0005198:structural molecule activity;
H4c14	H4 clustered histone 14	1.9	0.0005	0.0121	-	-	-

Ubxn11	UBX domain protein 11	1.8	0.0005	0.0114	-	GO:0005856:cytoskeleton;GO:0005737:cytoplasm;	-
Ak8	adenylate kinase 8	1.6	0.0003	0.0086	GO:0016310:phosphorylation;GO:0006139:nucleobase-containing compound metabolic process;GO:0021591:ventricular system development;	GO:0005829:cytosol;	GO:0004017:adenylate kinase activity;GO:0005524:ATP binding;GO:0004127:cytidylate kinase activity;
Hykk	hydroxylysine kinase 1	1.4	0.0005	0.0120	GO:0016310:phosphorylation;	GO:0005737:cytoplasm;	GO:0047992:hydroxylysine kinase activity;
Ggn	gametogenetin	1.4	0.0030	0.0441	GO:0006461:protein complex assembly;GO:0007283:spermatogenesis;GO:0008104:protein localization;GO:0030154:cell differentiation;GO:0007275:multicellular organismal development;GO:0044699:single-organism process;	GO:0005730:nucleolus;GO:0005635:nuclear envelope;GO:0016021:integral component of membrane;GO:0031410:cytoplasmic vesicle;GO:0048471:perinuclear region of cytoplasm;	GO:0046983:protein dimerization activity;
Avil	advillin	1.4	0.0005	0.0126	GO:0060271:cilium morphogenesis;GO:0010976:positive regulation of neuron projection development;GO:0051693:actin filament capping;GO:0006950:response to stress;	GO:0005856:cytoskeleton;GO:0042995:cell projection;GO:0005737:cytoplasm;	GO:0003779:actin binding;

Cd74	CD74 antigen (invariant polypeptide of major histocompatibility complex class II antigen-associated)	1.2	0.0001	0.0026	GO:0006461:protein complex assembly;GO:0002830:positive regulation of type 2 immune response;GO:0048146:positive regulation of fibroblast proliferation;GO:0002906:negative regulation of mature B cell apoptotic process;GO:0002606:positive regulation of dendritic cell antigen processing and presentation;GO:0070374:positive regulation of ERK1 and ERK2 cascade;GO:0045060:negative thymic T cell selection;GO:0051085:chaperone mediated protein folding requiring cofactor;GO:2000343:positive regulation of chemokine (C-X-C motif) ligand 2 production;GO:0019886:antigen processing and presentation of exogenous peptide antigen via MHC class II;GO:0016064:immunoglobulin mediated immune response;GO:0043518:negative regulation of DNA damage response, signal transduction by p53 class mediator;GO:0050731:positive regulation of peptidyl-tyrosine phosphorylation;GO:0030890:positive regulation of B cell proliferation;GO:0045582:positive	GO:0035693:NOS2-CD74 complex;GO:0005771:multivesicular body;GO:0005764:lysosome;GO:0005783:endoplasmic reticulum;GO:0035692:macrophage migration inhibitory factor receptor complex;GO:0005887:integral component of plasma membrane;GO:0005794:Golgi apparatus;GO:0009897:external side of plasma membrane;GO:0042613:MHC class II protein complex;	GO:0050998:nitric-oxide synthase binding;GO:0035718:macrophage migration inhibitory factor binding;GO:0042289:MHC class II protein binding;GO:0001540:beta-amyloid binding;GO:0004896:cytokine receptor activity;

					regulation of T cell differentiation;GO:0001516:prostaglandin biosynthetic process;GO:0045581:negative regulation of T cell differentiation;GO:0006886:intracellular protein transport;GO:0045089:positive regulation of innate immune response;GO:0060907:positive regulation of macrophage cytokine production;GO:0090023:positive regulation of neutrophil chemotaxis;GO:0001961:positive regulation of cytokine-mediated signaling pathway;GO:0045059:positive thymic T cell selection;GO:0000187:activation of MAPK activity;GO:0002792:negative regulation of peptide secretion;GO:0035691:macrophage migration inhibitory factor signaling pathway;GO:0002821:positive regulation of adaptive immune response;		
Pfn4	profilin family member 4	1.1	0.0029	0.0432	GO:0030036:actin cytoskeleton organization;	GO:0005856:cytoskeleton;GO:0005737:cytoplasm;	GO:0003779:actin binding;
Gsdmd	gasdermin D	-1.0	0.0022	0.0353	GO:0031668:cellular response to extracellular stimulus;	-	-

Mterf1b	mitochondrial transcription termination factor 1b	-1.1	0.0022	0.0363	GO:0006355:regulation of transcription, DNA-templated;GO:0032392:DNA geometric change;GO:0006393:termination of mitochondrial transcription;	GO:0042645:mitochondrial nucleoid;	GO:0003690:double-stranded DNA binding;
Pgf	placental growth factor	-1.1	0.0012	0.0232	GO:0051781:positive regulation of cell division;GO:0001938:positive regulation of endothelial cell proliferation;GO:0007186:G-protein coupled receptor signaling pathway;GO:0032496:response to lipopolysaccharide;GO:0060688:regulation of morphogenesis of a branching structure;GO:0043066:negative regulation of apoptotic process;GO:0031100:organ regeneration;GO:0045766:positive regulation of angiogenesis;GO:0002040:sprouting angiogenesis;GO:0001658:branching involved in ureteric bud morphogenesis;GO:0007565:female pregnancy;GO:0001666:response to hypoxia;GO:0042493:response to drug;GO:0030154:cell differentiation;GO:0032870:cellular response to hormone stimulus;	GO:0005615:extracellular space;GO:0016021:integral component of membrane;GO:0005737:cytoplasm;GO:0005886:plasma membrane;	GO:0004958:prostaglandin F receptor activity;GO:0008083:growth factor activity;GO:0005172:vascular endothelial growth factor receptor binding;GO:0046982:protein heterodimerization activity;GO:0042803:protein homodimerization activity;

Cracr2a	calcium release activated channel regulator 2A	-1.1	0.0000	0.0003	GO:0002115:store-operated calcium entry;GO:0051928:positive regulation of calcium ion transport;GO:0032237:activation of store-operated calcium channel activity;	GO:0005737:cytoplasm;	GO:0005509:calcium ion binding;
Ankrd2	ankyrin repeat domain 2 (stretch responsive muscle)	-1.2	0.0002	0.0049	GO:0035914:skeletal muscle cell differentiation;	GO:0005634:nucleus;GO:0031674:I band;	GO:0031432:titin binding;
Adam11	a disintegrin and metallopeptidase domain 11	-1.3	0.0004	0.0098	GO:0007229:integrin-mediated signaling pathway;GO:0006508:proteolysis;	GO:0016021:integral component of membrane;	GO:0004222:metalloendopeptidase activity;GO:0005515:protein binding;GO:0008270:zinc ion binding;
Col4a4	collagen type IV alpha 4	-1.4	0.0006	0.0138	GO:0032836:glomerular basement membrane development;	GO:0005587:collagen type IV trimer;GO:0005605:basal lamina;	GO:0005201:extracellular matrix structural constituent;
-	TEC	-1.5	0.0006	0.0128	-	-	-
Cpz	carboxypeptidase Z	-1.5	0.0021	0.0350	GO:0016055:Wnt signaling pathway;GO:0006508:proteolysis;GO:0016485:protein processing;	GO:0005578:proteinaceous extracellular matrix;	GO:0004181:metallocarboxypeptidase activity;GO:0008270:zinc ion binding;
Ramp3	receptor (calcitonin) activity modifying protein 3	-1.8	0.0021	0.0350	GO:0006816:calcium ion transport;GO:0001921:positive regulation of receptor recycling;GO:0045892:negative regulation of transcription, DNA-templated;GO:0006886:intracellular protein transport;GO:0031623:receptor internalization;GO:0072659:protein localization to plasma	GO:0005764:lysosome;GO:0016021:integral component of membrane;GO:0009986:cell surface;GO:0043235:receptor complex;GO:0005886:plasma membrane;	GO:0008565:protein transporter activity;GO:0015026:coreceptor activity;GO:0046982:protein heterodimerization activity;GO:0001664:G-protein coupled receptor binding;

					membrane;GO:0008277:regulation of G-protein coupled receptor protein signaling pathway;		
Mblac1	metallo-beta-lactamase domain containing 1	-1.8	0.0021	0.0350	-	-	GO:0016787: hydrolase activity;GO:0046872:metal ion binding;
Fam177a2	family with sequence similarity 177 member A2	-2.1	0.0000	0.0000	GO:0006614:SRP-dependent cotranslational protein targeting to membrane;	GO:0048500:signal recognition particle;	GO:0005525: GTP binding;GO:0003924:GTPase activity;GO:0008312:7S RNA binding;
Nr1i2	nuclear receptor subfamily 1 group I member 2	-2.4	0.0021	0.0352	GO:0045892:negative regulation of transcription, DNA-templated;GO:0042738:exogenous drug catabolic process;GO:0043401:steroid hormone mediated signaling pathway;GO:0006805:xenobiotic metabolic process;GO:0030522:intracellular receptor signaling pathway;GO:0045893:positive regulation of transcription, DNA-templated;GO:0046618:drug export;GO:0042908:	GO:0005634:nucleus;	GO:0005515: protein binding;GO:0008270:zinc ion binding;GO:0003707:steroid hormone receptor activity;GO:0008144:drug binding;GO:0004879:ligand-activated sequence-specific DNA binding RNA polymerase II transcription factor

					xenobiotic transport;		activity;GO:0043565:sequence-specific DNA binding;
Aass	aminoadipate-semialdehyde synthase	-2.5	0.0012	0.0238	GO:0033512:L-lysine catabolic process to acetyl-CoA via saccharopine;GO:0055114:oxidation-reduction process;GO:0006091:generation of precursor metabolites and energy;	GO:0005739:mitochondrion;	GO:0047130:saccharopine dehydrogenase (NADP+, L-lysine-forming) activity;GO:0000166:nucleotide binding;GO:0047131:saccharopine dehydrogenase (NAD+, L-glutamate-forming) activity;
Irs3	insulin receptor substrate 3	-2.7	0.0032	0.0465	-	GO:0005829:cytosol;GO:0005886:plasma membrane;GO:0043231:intracellular membrane-bounded organelle;	GO:0043548:phosphatidylinositol 3-kinase binding;GO:0005158:insulin receptor binding;

P2ry2A:H	purinergic receptor P2Y2 G-protein coupled 2	-2.8	0.0000	0.0005	GO:0035589:G-protein coupled purinergic nucleotide receptor signaling pathway;GO:0032722:positive regulation of chemokine production;GO:0032308:positive regulation of prostaglandin secretion;GO:0042312:regulation of vasodilation;GO:0007204:positive regulation of cytosolic calcium ion concentration;GO:0060406:positive regulation of penile erection;GO:0030279:negative regulation of ossification;GO:0070257:positive regulation of mucus secretion;GO:0010976:positive regulation of neuron projection development;GO:0019233:sensory perception of pain;GO:0014911:positive regulation of smooth muscle cell migration;	GO:0016323:basolateral plasma membrane;GO:0005764:lysosome;GO:0016021:integral component of membrane;GO:0005769:early endosome;GO:0016324:apical plasma membrane;GO:0005739:mitochondrion;	GO:0045030:UTP-activated nucleotide receptor activity;GO:0045028:G-protein coupled purinergic nucleotide receptor activity;GO:0005524:ATP binding;GO:0031686:A1 adenosine receptor binding;
Col14a1	collagen%2C type XIV alpha 1	-3.2	0.0001	0.0031	GO:0048873:homeostasis of number of cells within a tissue;GO:0007155:cell adhesion;GO:0030199:collagen fibril organization;GO:0061050:regulation of cell growth involved in cardiac muscle cell development;GO:0003229:ventricular cardiac muscle tissue development;	GO:0005581:collagen trimer;GO:0005615:extracellular space;GO:0005614:interstitial matrix;	-
Snx22	sorting nexin 22	-3.5	0.0022	0.0363	-	-	-

Mir210	microRNA 210	-4.2	0.0032	0.0462	-	-	-
U5	U5 spliceosomal RNA	-4.2	0.0032	0.0462	-	-	-

**Note: This supplementary dataset represents an exploratory pilot screening framework derived from a single-replicate high-throughput run (p<0.05). All listed transcripts are explicitly designated as candidate baseline discovery targets intended to guide subsequent downstream functional validation assays (e.g. qPCR, targeted genetic knockdown/overexpression models, or downstream Western blotting)*
